# Therapeutic Notes

**Published:** 1913-12

**Authors:** J. M. Fortescue-Brickdale


### Therapeutic Notes.

### GALACTOGOGUES, OLD AND NEW.

It is the very general opinion of experienced physicians that little can be done to increase the secretion of milk in nursing mothers beyond such general measures as are included in the diet and hygiene of the puerperium. Nor is this surprising, considering how greatly the secretion of the lacteal glands differs from that of all the other glands in the body, and what a tremendous metabolic activity is implied in the elaboration of a fluid so rich in organic substances, fatty, albuminous and carbohydrates.

Three of the larger modern text-books on pharmacology and therapeutics do not include the word galactogogue in their indices. Older works mention two, alcohol and pilocarpine. The administration of alcohol is in many cases undesirable, and in all inefficacious, except possibly in a very secondary way. Pilocarpine is also discredited; if it has any action at all on the mammary glands it is most uncertain, and it has never been shown to increase the solid constituents of the milk. Of late years, however, three substances have been introduced which have been stated to act as galactogogues, and in view of the importance of breast-feeding, it is desirable that their possible value should be carefully considered, and a trial given to such of them as may, *prima facie*, seem likely to be of value.

The first of these is known under the trade name of *Galegol*, and is obtained from the plant *Galega officinalis*, by extraction at a low temperature. The plant is one of the large family of leguminosæ, and is used in Southern Europe for feeding milch cows. Galegol consists of a brown granular soluble substance, the taste of which is not unpleasant, and it is administered in doses of two teaspoonfuls to one tablespoonful *per diem*, mixed in milk or some other convenient vehicle, at a temperature not above 98.6° F. It does not appear to be uniformly successful, and in cases in which the quantity of milk increased, it is difficult to say how far this was due to other factors. At any rate, in some cases, in which the drug was suddenly discontinued, no decrease in the amount of milk was observed.

The next substance goes by the name of *Lactagol,* and is an extract of cotton seed (or hemp seed), the active principle of which is said to be the vegetable protein called Edestine. Cotton seed has for some time been used in feeding milch cows, but is unsuitable for medication in the human subject, owing to the presence of various indigestible and irritating ingredients. In the process of manufacture the seeds are first finely powdered, and the oily and resinous portions removed by extraction with ether or benzine. An infusion of the residue contains the active principle, which is thus freed from colouring matter and other impurities. The watery substance is evaporated *in vacuo,* and then ground into a fine brown powder, consisting almost entirely of protein, which swells up on boiling, but is not soluble in water. The dose is one teaspoonful stirred into a paste with cold milk, to which warm, but not hot, milk, or some other convenient vehicle can be added. It may be given three times daily. A good many favourable clinical reports have been made on *Lactagol,* and it certainly seems worthy of an extended trial, especially as it is apparently quite harmless.

The third substance is an extract of the posterior portion of the pituitary gland, which may be injected subcutaneously, or into the deeper tissues. Professor Schäfer's earlier experiments on cats showed that a considerable flow of milk followed the injection of pituitary extract, but further investigations seem to show that its value is not so great as at first appeared. It is apparently true that a flow of milk can be excited by pituitary extract in women ; thus Mackenzie reported that in a woman who had only one breast available for lactation, an injection caused a flow of 100 c.c. of milk one hour after the breast had been pumped dry. But the very careful experiments of Gavin on cows led him to the conclusion that although doses of pituitary extract caused an increased collection of milk in the lower part of the udder, there was no increase in the total quantity secreted *per diem,* and no improvement in the quality of the yield. A single case in the human subject reported by Sumpter seems to corroborate this result. The experiment was made on a young married woman aged 28, who was nursing her second child. The milk, after five months' lactation, was beginning to fail. Four injections of 1 to 1.5 c.c. of pituitary extract, each c.c. of which represented 0.2 gram of fresh posterior lobe, was injected at intervals of two or three days. Shortly, the result was some immediate effect in increasing the flow, but longer intervals of rest between the feedings became necessary, and the gradual decrease in the total amount of the secretion continued. Until further evidence, therefore, is available, pituitary extract cannot be regarded as a practical galactogogue. Sumpter also experimented on the same subject with extract of the corpus luteum of the sheep, three injections of 2 c.c. (representing 0.1 gram of the dry substance in each c.c.) being given without any marked effect. The full reports of these experiments will be found in the *Quarterly Journal of Experimental Physiology* for the present year.

J. M. FORTESCUE-BRICKDALE.

